# Homeodomain Interacting Protein Kinase 2: A Target for Alzheimer's Beta Amyloid Leading to Misfolded p53 and Inappropriate Cell Survival

**DOI:** 10.1371/journal.pone.0010171

**Published:** 2010-04-14

**Authors:** Cristina Lanni, Lavinia Nardinocchi, Rosa Puca, Serena Stanga, Daniela Uberti, Maurizio Memo, Stefano Govoni, Gabriella D'Orazi, Marco Racchi

**Affiliations:** 1 Department of Experimental and Applied Pharmacology, Centre of Excellence in Applied Biology, University of Pavia, Pavia, Italy; 2 Molecular Oncogenesis Laboratory, Department of Experimental Oncology, National Cancer Institute Regina Elena, Rome, Italy; 3 Department of Oncology and Neurosciences, University “G. d'Annunzio”, Chieti, Italy; 4 Department of Biomedical Sciences and Biotechnologies, University of Brescia, Brescia, Italy; Mental Health Research Institute of Victoria, Australia

## Abstract

**Background:**

Homeodomain interacting protein kinase 2 (HIPK2) is an evolutionary conserved serine/threonine kinase whose activity is fundamental in maintaining wild-type p53 function, thereby controlling the destiny of cells when exposed to DNA damaging agents. We recently reported an altered conformational state of p53 in tissues from patients with Alzheimer's Disease (AD) that led to an impaired and dysfunctional response to stressors.

**Methodology/Principal Findings:**

Here we examined the molecular mechanisms underlying the impairment of p53 activity in two cellular models, HEK-293 cells overexpressing the amyloid precursor protein and fibroblasts from AD patients, starting from recent findings showing that p53 conformation may be regulated by HIPK2. We demonstrated that beta-amyloid 1–40 induces HIPK2 degradation and alters HIPK2 binding activity to DNA, in turn regulating the p53 conformational state and vulnerability to a noxious stimulus. Expression of HIPK2 was analysed by western blot experiments, whereas HIPK2 DNA binding was examined by chromatin immunoprecipitation analysis. In particular, we evaluated the recruitment of HIPK2 onto some target promoters, including hypoxia inducible factor-1α and metallothionein 2A.

**Conclusions/Significance:**

These results support the existence of a novel amyloid-based pathogenetic mechanism in AD potentially leading to the survival of injured dysfunctional cells.

## Introduction

The protein p53 responds to a variety of cellular stresses and may induce cell cycle arrest or apoptosis In fact, by differential activation of a large number of target genes and by mitochondrial functions, p53 is able to sense the intensity of the damage and modulate biological responses that can range from transient growth arrest to permanent replicative senescence or apoptosis [1]. The induction of p53 transcriptional activity depends mainly on posttranslational modifications together with protein/protein interaction [2]. Another important mechanism that controls p53 function is its conformational stability since p53 is an intrinsically unstable protein whose structure includes one zinc atom as an important co-factor for DNA-binding activity *in vitro* and *in vivo* [3], [4]. An increased content of an unfolded p53 protein isoform [5]–[7] has been reported in numerous tumour cells where p53 harboured different gene point mutations. On the other hand, conformational changes of p53 towards unfolded isoforms are not only associated with gene mutations, but post transcriptional modifications can affect p53 tertiary structure. It is worth noting that in cellular models of Alzheimer's Disease (AD), p53 was found to be conformationally altered, making these cells less vulnerable to stressors or genotoxic insults [5]–[7]. When investigating the mechanism of this alteration, we found that the exposure to nanomolar concentrations of beta-amyloid (Aβ) 1-40 peptide was responsible for the increased content of unfolded p53 protein [8]. One of the activators that induces p53 posttranslational modification and wild-type conformational stability is homeodomain interacting protein kinase 2 (HIPK2), an evolutionary conserved serine/threonine kinase able to regulate gene expression by the phosphorylation of transcription factors and accessory components of the transcription machinery. HIPK2 is activated in response to DNA damaging agents or morphogenic signals, thus playing a key role in differentiation, development or apoptosis [for a review see refs [9] and [10]]. HIPK2 interacts physically and functionally with p53 and specifically phosphorylates p53 at serine 46 (Ser46) in response to severe DNA damage, regulating p53-induced apoptosis [11], [12]. In addition, it has recently been shown that HIPK2 depletion results in p53 protein misfolding, changing the wild-type conformation to a conformationally altered status with subsequent abolishment of wild type p53 DNA binding and transcriptional activity. This can be restored with zinc supplementation [13].

Our purpose was to evaluate whether the altered conformational state of p53, observed in AD cells, was dependent on an impaired HIPK2 function and, by the use of two cellular models of AD, to define whether a deregulation of HIPK2 is involved in AD pathogenesis. Furthermore, since AD is characterized by an aberrant metabolism of the amyloid precursor protein (APP), in turn resulting in an aberrant production of Aβ peptides [14], our intent was to investigate the effect of Aβ peptides on HIPK2 expression and DNA-binding activity. The results presented here may define a hierarchical scale of events related to Aβ activities and eventually lead to a better understanding of AD pathogenesis.

## Results

### Aβ is responsible for HIPK2 deregulation

**Figure 1:**
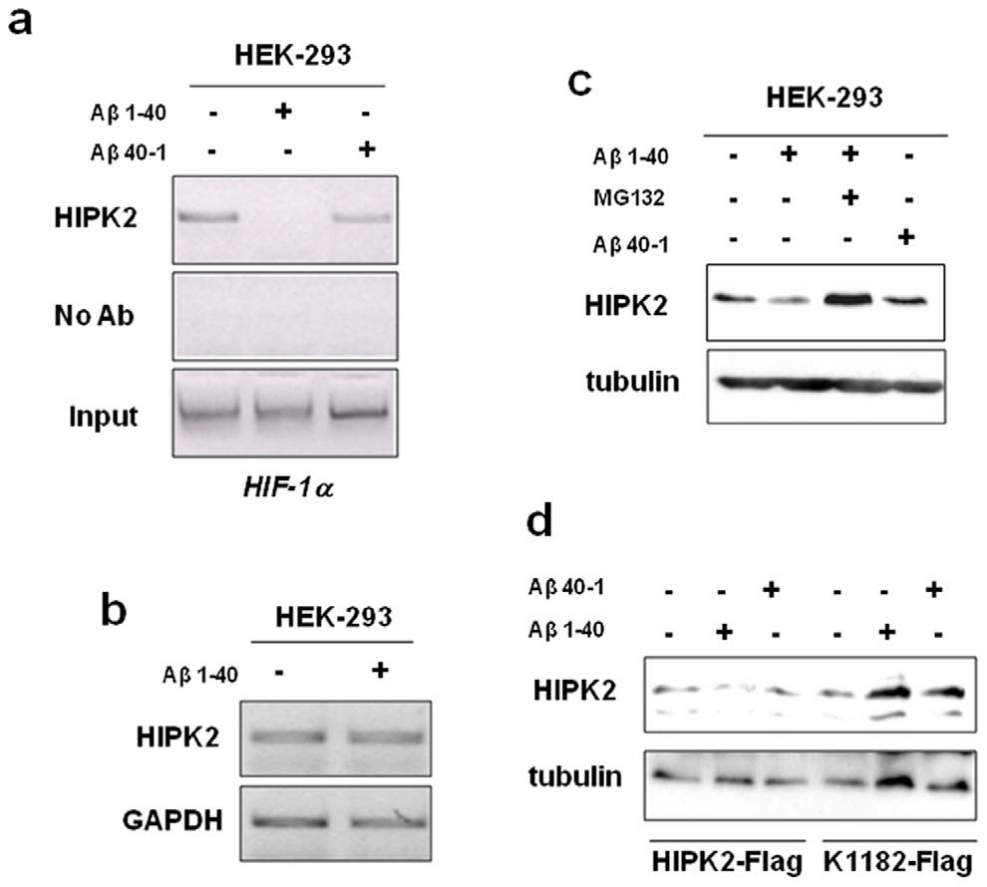
Aβ is responsible for HIPK2 deregulation. (**a**) ChIP experiments were performed with anti-HIPK2 antibody on HEK-293 cells treated with 10 nM Aβ 1-40 or Aβ 40-1 for 48 h. PCR analyses were performed on the immunoprecipitated DNA samples using specific primers for the human HIF-1α promoter. A sample representing linear amplification of the total input chromatin (Input) was included as control. Additional controls included immunoprecipitation performed with non-specific immunoglobulins (no Ab). A representative experiment of three independent ones was shown. (**b**) HIPK2 mRNA expression was determined in HEK-293 cells, treated with 10 nM Aβ 1-40 for 48 h, by reverse-transcriptase (RT)-PCR. GAPDH was used as loading control. (**c**) Total cell extracts of HEK-293 cells treated with 10 nM Aβ 1-40 for 48 h in the absence or presence of 10 µmol/L MG132 for 6 h were analysed for HIPK2 expression. Anti-tubulin was used as protein loading control. (**d**) HEK-293 cells were transfected with HIPK2-Flag and the degradation-resistant HIPK2-K1182R mutant expression vector. After transfection, cells were trypsinized, replated in triplicate and treated with 10 nM Aβ 1-40 or the reverse peptide Aβ 40-1 for 48 h. Total cell extracts were analysed by Western immunoblotting with anti-Flag antibody. Anti-tubulin was used as protein loading control.

We first investigated whether HIPK2 DNA-binding to target promoters was somehow compromised by nanomolar concentrations of soluble Aβ peptides. To this aim, we treated HEK-293 cells with soluble Aβ 1-40 at the concentration of 10 nM for 48 h. As we have previously reported, this enters the cells and induces conformational changes in p53 protein [5]. Subsequently chromatin immunoprecipitation (ChIP) experiments were performed in order to evaluate the integrity of interaction between HIPK2 and the hypoxia inducible factor-1α (HIF-1α) promoter, as recently reported [15]. As shown in Figure 1a, HIPK2 binding to HIF-1α promoter was eliminated by Aβ 1-40 treatment, whereas treatment with the reverse peptide Aβ 40-1 failed to do so. We further evaluated Aβ 1-40-induced HIPK2 down-regulation and mRNA expression was assessed in HEK-293 cells after treatment with Aβ 1-40. As shown in Figure 1b, the analysis of RT-PCR following normalization to GAPDH expression revealed that no differences in HIPK2 mRNA expression were observed when comparing treated to untreated HEK-293 cells. Next, HIPK2 protein expression was evaluated by Western immunoblotting. As shown in Figure 1c, Aβ 1-40 treatment reduced HIPK2 protein levels in HEK-293 cells, compared to vehicle or Aβ 40-1 treatment. Cell treatment with proteasome inhibitor MG132 strongly reduced Aβ 1-40-dependent HIPK2 down regulation, increasing HIPK2 protein levels to an even larger extent when compared to control cells (Figure 1c), suggesting that additional proteasomal degradation processes besides that induced by Aβ 1-40 are likely involved in HIPK2 degradation. Then HEK-293 cells were transfected with HIPK2-Flag and the degradation-resistant HIPK2-K1182R mutant expression vectors. Subsequently cells were treated with Aβ 1-40 or the reverse inactive Aβ 40-1 peptide. As shown in Figure 1d, HIPK2-Flag expression was down regulated by Aβ 1-40 while the reverse peptide was not effective, as reported above for endogenous HIPK2 (Figure 1c); Aβ 1-40 did not affect the expression of the K1182 mutant (Figure 1d). Altogether, these results show that nanomolar concentrations of soluble Aβ can impair HIPK2 binding to DNA, likely through activation of proteasomal degradation, as assessed by the use of the proteasome inhibitor MG132.

### Endogenous products of APP metabolism negatively affect HIPK2/DNA binding activity

**Figure 2:**
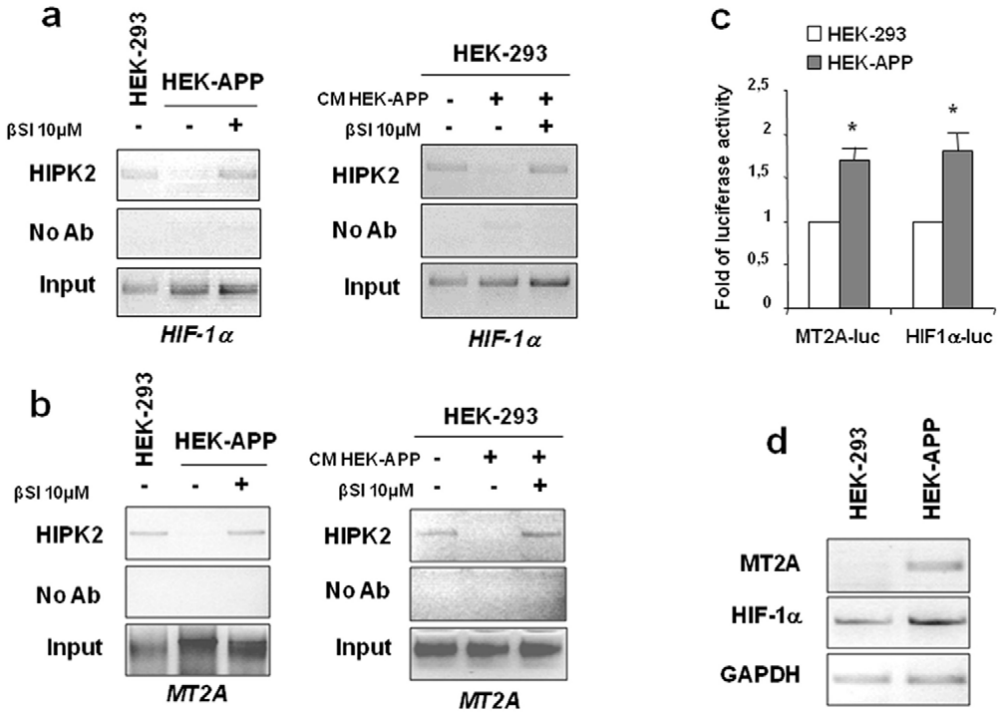
Endogenous products of APP metabolism negatively affect HIPK2 DNA-binding activity. (**a**) ChIP experiments were performed with anti-HIPK2 antibody on HEK-293 and HEK-APP cells that were also treated with β-secretase inhibitor at 1 µmol/L for 48 h and on HEK-293 treated with conditioned medium from HEK-APP cells for 48 h in the absence or presence of β-secretase inhibitor; PCR analyses were performed on the immunoprecipitated DNA samples using specific primers for the human HIF-1α promoter as shown in Figure 1. (**b**) ChIP experiments were performed with anti-HIPK2 antibody on HEK-293 and HEK-APP cells that were also treated with β-secretase inhibitor at 1 µmol/L for 4 8h and on HEK-293 treated with conditioned medium from HEK-APP cells for 48 h in the absence or presence of β-secretase inhibitor; PCR analyses were performed on the immunoprecipitated DNA samples using specific primers for the MT2A promoter. (**c**) HEK-293 and HEK-APP cells were transfected with MT2A-luc and HIF-1α-luc reporter construct and luciferase activity was measured 36 h after transfection. Results normalized to β-galactosidase activity are presented as fold of induction of luciferase activity ±S.D. At least three independent experiments performed in duplicate. * *p*<0.01 (Student *t*-test). (**d**) MT2A and HIF-1α mRNA expression was determined in HEK-APP compared to HEK-293 cells by reverse-transcriptase (RT)-PCR. GAPDH was used as loading control.

To acquire more insight into the contribution of the different APP processing products to HIPK2 deregulation, we used HEK-293 cells stably transfected with wild type APP751 (HEK-APP) that express high levels of full length APP in comparison with HEK-293 cells [5]. In particular, HEK-APP cells produced and released elevated amounts of Aβ peptides (see Table 1), where Aβ 1-40 was the more abundant isoform (700 pg/mg protein in cellular extracts and 200 pg/mL in the conditioned medium after 48 hrs) than Aβ 1-42 (78 pg/mg in cellular extracts and 42 pg/mL in the conditioned medium after 48 hrs). In order to prevent amyloidogenic APP metabolism, HEK-APP cells were then treated with β secretase inhibitor (βSI); Aβ 1-40 and Aβ 1-42 levels were then measured in both cellular extracts and medium after β secretase inhibition. As shown in Table 1, inhibition of APP amyloidogenic pathway induced by β secretase inhibitor prevented the formation of Aβ 1-40 and Aβ 1-42 in both the cellular extract and in the medium. HIPK2 binding to HIF-1α promoter was then evaluated in HEK-293 cells and HEK-APP cells that were also treated with the βSI. As shown in Figure 2a (left panel), HIPK2 was easily detected in the HIF-1α promoter in HEK-293 control cells while the recruitment was abolished in HEK-APP cells. Beta-secretase inhibitor treatment enhanced HIPK2 recruitment onto HIF-1α promoter (Figure 2a, left panel), strongly suggesting that APP amyloidogenic metabolites may indeed affect HIPK2 DNA-binding activity. We then tested the ability of the conditioned medium of HEK-APP cells to inhibit HIPK2 DNA-binding. To this aim, HEK-293 cells were cultured with conditioned medium of HEK-APP cells that, as shown in Figure 2a (right panel), abolished HIPK2 binding to HIF-1α promoter. In agreement with our hypothesis, β-secretase inhibitor treatment counteracted the APP-conditioned medium ability to inhibit HIPK2 binding to DNA and re-established HIPK2 recruitment onto HIF-1α promoter (Figure 2a, right panel).

**Table 1:** Levels of Aβ 1-40 and Aβ 1-42 after treatment with β secretase inhibitor.

	Cell extracts (pg/mg protein)	Medium (pg/ml)
**Aβ 1-40 levels**	700±6.28	200±9.12
**Aβ 1-42 levels**	78±5.18	42±5.21
**Aβ 1-40 levels after βSI**	480±3.17*	128±4.32*
**Aβ 1-42 levels after βSI**	42±2.12#	28±1.15#

Levels of Aβ 1-40 and Aβ 1-42 peptides were measured with a commercial ELISA kit in the cellular extracts and conditioned media of HEK-APP cells untreated or treated with β secretase inhibitor at 1 µmol/L for 48 hours. Results are representative of at least three independent experiments ± S.E.M.

* p<0.001 βSI treatment vs corresponding control.

# p<0.01 βSI treatment vs corresponding control.

A mechanism through which HIPK2 deregulation may affect p53 conformation could be through metallothionein 2A (MT2A). Metallothioneins can act as potent chelators in removing zinc from p53 *in vitro* and may modulate p53 transcriptional activity [16]. In particular, HIPK2 depletion has been observed to induce MT2A upregulation, whose inhibition by siRNA restored p53 wild-conformation and transcriptional activity [17]. These findings suggest that HIPK2 plays a critical role in maintaining p53 wild-type conformation for DNA binding and transcriptional activity indirectly through MT2A down regulation. Hence, we investigated whether HIPK2 activity to bind MT2A target promoter was somehow compromised in HEK-APP. As shown in Figure 2b (left panel), ChIP assay showed that the HIPK2 recruitment onto MT2A promoter was hampered in HEK-APP cells in comparison with HEK-293 control cells, whereas it was recovered by treatment with β secretase inhibitor. Furthermore, when HEK-293 cells were treated with conditioned medium of HEK-APP cells, we observed an elimination of HIPK2 binding to MT2A promoter (Figure 2b, right panel). Parallel to data on HIF-1α, β-secretase inhibitor treatment re-established HIPK2 recruitment onto MT2A promoter thus counteracting the APP-conditioned medium ability to affect HIPK2 binding to DNA (Figure 2b, right panel). These data were supported by the increased HIF-1α-luc and MT2A-luc activities in HEK-APP cells compared to the HEK-293 counterparts (Figure 2c). In agreement, MT2A and HIF-1α mRNA were induced in HEK-APP compared to HEK-293 cells, although to a different extent (Figure 2d). These data suggest that impaired HIPK2 binding to DNA in AD cells correlated with increased HIF-1α and MT2A expression.

### p53 transcriptional activity is restored by zinc

**Figure 3:**
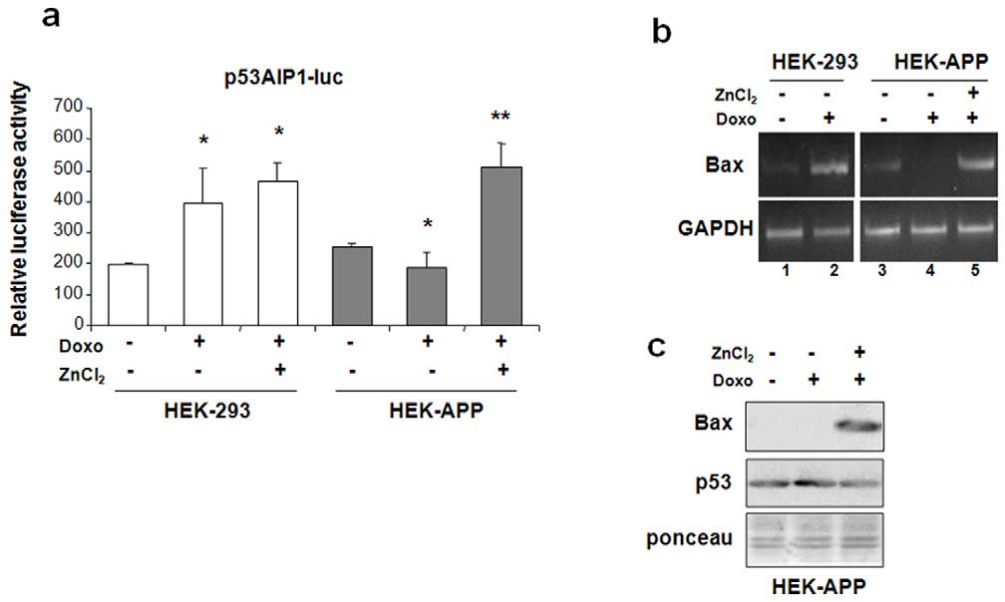
Zinc supplementation to HEK-APP restores p53 pro-apoptotic transcriptional activity. (**a**) HEK-293 and HEK-APP cells were transfected with p53AIP1-luc reporter construct and 24 h after transfection treated with doxorubicin (3.4 µM) and zinc (100 µM) for 24 h before luciferase activity was assayed. Results normalized to β-galactosidase activity are shown as relative luciferase activity ±S.D. At least three independent experiments performed in duplicate. * *p*<0.05 vs HEK-293 or HEK-APP; ** *p*<0.01 vs HEK-APP (Bonferroni Multiple Comparison test). (**b**) Bax mRNA expression was determined in HEK-APP compared to HEK-293 cells by reverse-transcriptase (RT)-PCR after treatment with doxorubicin (3.4 µM) and zinc (100 µM) for 24 h. GAPDH was used as loading control. (**c**) Total cell extracts of HEK-APP cells treated with doxorubicin (3.4 µM) and zinc (100 µM) for 24 h were analysed for Bax and p53 expression. Protein loading control was shown as Ponceau staining.

Following the data on MT2A overexpression, we wanted to evaluate whether the p53 dysfunction, related to Aβ exposure in our experimental models, could be restored by zinc supplementation. Thus, p53 transcriptional activity was evaluated by luciferase assay of the p53AIP1-luc apoptotic promoter [18]. HEK-APP cells and the control counterparts were transiently transfected with the p53AIP1-luc reporter plasmid and 24 hrs later treated with 3.4 µM doxorubicin, a cytotoxic agent able to induce DNA damage and apoptosis in a p53-dependent manner [19]. As shown in Figure 3a, p53AIP1-luciferase activity was induced by doxorubicin treatment in HEK-293 cells, whereas it was significantly impaired in HEK-APP cells. Zinc supplementation to HEK-APP cells restored endogenous p53 ability to activate p53AIP1-luciferase reporter in response to doxorubicin (Figure 3a), while it only slightly increased p53 transcriptional activity in HEK-293 cells. Next, analysis of mRNA showed that the doxorubicin-induced p53 apoptotic gene transcription (i.e., Bax gene) in HEK-293 cells was impaired after stable transfection of APP751 (HEK-APP) (Figure 3b, compare lane 4 *vs* lane 2). In agreement with our hypothesis, zinc supplementation to HEK-APP cells restored drug-induced Bax transcription (Figure 3b, compare lane 4 *vs* lane 5). Finally, Western immunoblotting showed doxorubicin-induced Bax expression in HEK-APP cells only after zinc supplementation (Figure 3c). These data suggest that the Aβ-inhibited wild-type 53 apoptotic transcriptional activity, in response to drug, was reactivated by zinc.

### AD fibroblasts show conformationally altered p53 protein and a reduced HIPK2 DNA-binding activity that are restored by zinc

**Figure 4:**
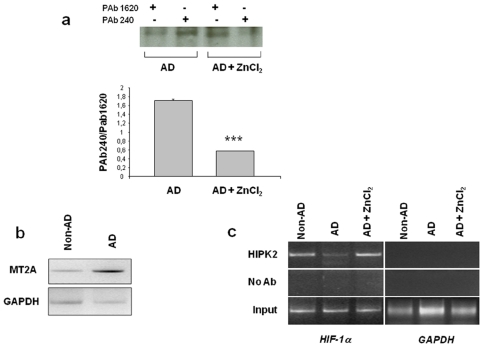
Fibroblasts from Alzheimer patients show misfolded p53 with increased MT2A expression and reduced HIPK2 binding to DNA. (**a, upper panel**) Equal amounts of total cell extracts from fibroblasts derived from AD patients were treated with 100 µM zinc for 16 h and then immunoprecipitated with anti-p53 conformational antibodies, PAb1620 for wild-type conformation and Pab240 for mutant-like conformation. Immunoprecipitates were analysed by Western immunoblotting with the polyclonal anti-p53 antibody. A representative experiment of three independent ones was shown. (**a, lower panel**) Densitometric analysis of immunoprecipitated p53 as above, showing reduction of PAb240 mutant-like conformation after zinc treatment. *** *p*<0.0001 vs AD (Student *t*-test). (**b**) MT2A expression was determined in fibroblasts derived from AD patients compared to non-AD patients by reverse-transcriptase (RT)-PCR. GAPDH was used as loading control. (**c**) ChIP experiments were performed with anti-HIPK2 antibody on fibroblasts from AD and non-AD patients. PCR analyses were performed on the immunoprecipitated DNA samples using specific primers for the human HIF-1α promoter. A sample representing linear amplification of the total input chromatin (Input) was included as control. Additional controls included immunoprecipitation performed with non-specific immunoglobulins (no Ab). A representative experiment of three independent experiments was shown. Amplification of GAPDH promoter was used as control of HIPK2 binding specificity to the HIF-1α promoter.

The conformational status of p53 was analyzed in fibroblasts derived from AD and non-AD subjects by immunoprecipitation technique using two conformational-specific antibodies, PAb1620 and PAb240, which discriminate folded versus unfolded p53 tertiary structure [16]. Fibroblasts from AD patients from our cell repository express high levels of unfolded p53, as shown by Uberti et al. [7] and replicated in the two representative cell lines used in the current experimental setting (Figure 4a). Zinc supplementation to AD fibroblasts strongly reduced p53 mutant-like conformation (Figure 4a, upper panel), as is also shown by quantitative analysis of p53 immunoprecipitates (Figure 4a, lower panel). Analysis of mRNA showed that MT2A expression was upregulated in AD cells (Figure 4b), suggesting that HIPK2 deregulation might be involved in p53 misfolding in AD cells, likely through MT2A upregulation, as previously shown [17]. As on HEK-APP cells, we then investigated whether HIPK2 activity to bind target promoters was somehow compromised in AD fibroblasts. As shown in Figure 4c, ChIP assay showed that the HIPK2 recruitment onto HIF-1α promoter was present in fibroblasts from control subjects, whereas it was hampered in AD cells. Of note, zinc treatment to AD fibroblasts restored HIPK2 binding activity to DNA, likely counteracting the proteasomal degradation, as previously reported [20] and thus suggesting that zinc was able to affect the dual role of HIPK2 as DNA co-repressor and as p53 activator.

## Discussion

For the first time, we can describe a link between Aβ, AD-related conformationally altered p53 and HIPK2, a transcriptional co-repressor and activator of p53 apoptotic function. We previously demonstrated the existence of an unfolded state of p53 protein in fibroblasts from AD patients that led to an impaired and dysfunctional response to stressor [6], [21]. Here we examined the molecular mechanisms underlying the impairment of p53 activity in two cellular models, HEK-293 cells overexpressing the amyloid precursor protein and fibroblasts from AD patients, starting from recent findings showing that p53 conformation may be regulated by HIPK2 [13]. Our data suggest that Aβ peptides may be responsible for HIPK2 deregulation. This is supported by the observation that Aβ peptides down-regulated HIPK2 expression via proteasomal degradation (Figure 1c and 1d) leading to HIPK2 disappearance from target promoters such as HIF-1α and MT2A (Figure 1a, 2a and 2b). In agreement, HIF-1α and MT2A mRNA upregulation was found in HEK-APP cells that overexpress APP751 (Figure 2d). The induction of MT2A, depending on HIPK2 knockdown has been reported to be responsible for p53 misfolding and inhibition of p53 transcriptional activity [16]; therefore, the present data suggest that HIPK2 deregulation in HEK-APP cells and fibroblasts from AD patients might be involved in p53 misfolding, most likely through MT2A upregulation.

Attempting to better investigate the contribution of APP metabolic products in the modulation of HIPK2 expression and change in p53 conformational state, we then used HEK cells with wild-type APP able to generate high levels of Aβ 1-40 and Aβ 1-42 both intracellularly and secreted in the medium, with Aβ 1-40 about 10 times more abundant than Aβ 1-42 [5]. We found that reducing APP amyloidogenic metabolism by treating HEK-APP cells with a β-secretase inhibitor prevented the deregulation of HIPK2 (Figure 2a, 2b) and likely the generation of the unfolded p53 isoform. Interestingly, we previously demonstrated that α-secretase inhibitor did not affect unfolded p53 isoform and did not modify the cellular response to doxorubicin [5]. It is worthy to note that the conditioned medium of HEK-APP cells was able to affect HEK-293 cells recapitulating the HEK-APP phenotype, at least in terms of HIPK2 DNA-binding and altered p53 conformational changes. The Aβ peptides released in the media by HEK-APP appeared to trigger such effects. In fact, the conditioned media of HEK-APP cells pretreated with β secretase inhibitor, were unable to inhibit HIPK2 binding to HIF-1α and MT2A promoters, in HEK-293 cells (Figure 2b).

HIPK2 has been shown to be down-regulated during hypoxia by Siah2-induced proteasomal degradation [22]. Moreover, HIPK2 impairment during hypoxia induces de-repression of target genes such as HIF-1α, and inhibition of p53 activity [20]. Zinc supplementation to hypoxia-treated cells restores HIPK2 stability and binding to HIF-1α promoter, rescuing also the p53 apoptotic transcriptional activity [20]. Therefore, discovering the mechanisms of HIPK2 inhibition and the ways to manipulate HIPK2 activity is an interesting option to affect several biological pathways [23]. Here we showed a novel mechanism of HIPK2 down-regulation mediated by Aβ, likely through activation of proteasomal degradation. HIPK2 is an unstable protein that is degraded via the proteasome pathway induced by several E3 protein ligases [24], although the molecular mechanisms underlying HIPK2 proteasomal degradation in conditions related to Aβ-exposure deserve further studies.

The deregulation of HIPK2 function was further confirmed in fibroblasts from sporadic AD subjects, an extra-neuronal model showing a number of abnormalities in metabolic and biochemical processes, with some of them mirroring events that occur in the AD brain [25]. We found that fibroblasts from AD patients are characterized by a decreased HIPK2 DNA-binding activity, besides showing a conformationally altered p53. Since one of the features that distinguishes AD from non-AD fibroblasts is a defective non-amyloidogenic APP processing, likely favouring an aberrant Aβ peptides production [26], these data suggest that this abnormality may be, at least in part, responsible for altered HIPK2 binding to promoters. HIPK2 is ubiquitously expressed and has been found in developing neurons [27]. HIPK2 overexpression suppresses Brn3a-dependent transcription of *brn3a*, *trkA* and *bcl-x_L_* resulting in apoptotic cell death in cultured sensory neurons [27], [28]. Moreover, HIPK2 is an important component in the TGFβ signalling pathway that regulates the survival of midbrain dopamine neurons, as suggested by HIPK2 knock-out mice [28], [29]. Interestingly, it has been recently shown that HIPK2 is required for the trakA to p75^NTR^ transition that leads to increased generation of Aβ that accompanies aging [30], suggesting a regulatory loop that tends to inhibit HIPK2 during aging contributing to AD.

The impairment of HIPK2 function, by shifting p53 protein structure from a wild-type to a conformationally altered phenotype, should increase the threshold to a noxious stimulus, as reported [13]. Therefore, we tested, in HEK-APP cells, whether the reduced efficacy of doxorubicin-induced apoptosis was due to an impairment of p53 apoptotic activity. In agreement with our hypothesis, we found that these cells showed a reduced HIPK2 DNA binding activity and Bax transcription in response to doxorubicin that was rescued by zinc (Figure 3). Zinc has been reported to restore p53 function in HIPK2 depleted cells [13], [17]. The capability of zinc to act in cells is ascribed to the existence of zinc transporters, that are required to convey this ion across cellular membranes, since zinc is unable to passively diffuse across cell membranes [31]. The use of zinc for AD treatment is controversial, since several recent works reported the capability of zinc to cause the precipitation of Aβ into nonfibrillar amorphous aggregates [32]. However, in our experiments we speculate that the capability of zinc supplementation to restore the Aβ 1-40-inhibited HIPK2 DNA-binding appears not to be associated to a metal ion-induced precipitation of the synthetic peptide but rather to counteracting a degradation mechanism. This is supported by the observations in fibroblasts from AD patients, in which zinc treatment was able to rescue HIPK2 binding to its target promoters. However, we could also not exclude the hypothesis that zinc may be able to withdraw the synthetic peptide from cellular environment thus modulating its interaction.

In summary, we hypothesize that low amounts of soluble Aβ, not resulting in cellular toxicity, may be responsible for important modulatory effects at cellular level before triggering the amyloidogenic cascade. For the first time we found that one of these modulatory effects may be the inhibition of HIPK2 activity, with MT2A upregulation, in turn responsible for the induction of an altered conformational state of p53. As a result of this conformational change, p53 lost its transcriptional activity and was unable to properly activate an apoptotic program when cells were exposed to a noxious stimulus. Altogether, Aβ-induced HIPK2 depletion and unfolded p53 may contribute to AD pathogenesis leading to dysfunctional cells. The definition of this new target is useful to help characterize the hierarchical scale of events driven by beta-amyloid so as to better understand the pathogenesis of AD. Furthermore, the recognition of HIPK2 as new target of the effect of Aβ could suggest a new putative functional biomarker useful in addressing new therapeutic strategies.

## Materials and Methods

### Reagents and cell treatments

All culture media, supplements and Foetal Bovine Serum (FBS) were obtained from Euroclone (Life Science Division, Milan, Italy). Electrophoresis reagents were obtained from Bio-Rad (Hercules, CA, USA). All other reagents were of the highest grade available and were purchased from Sigma Chemical Co. (St. Louis, MO, USA) unless otherwise indicated. Amyloid-β (Aβ) peptide 1-40 and Aβ 40-1 reverse peptide were solubilised in DMSO at the concentration of 100 µM and frozen in stock aliquots that were diluted at the final concentration of 10 nM prior to use. For each experimental setting, one aliquot of the stock was thawed out and diluted at the final concentration of 10 nM to minimize peptide damage due to repeated freeze and thaw. The Aβ concentration was chosen following dose response experiments (data not shown) where maximal modulation of p53 structure and its transcriptional activity [5] was obtained at 10 nM. All the experiments performed with Aβ were made in 1% of serum. Doxorubicin was solubilised in H_2_O at the concentration of 10 mM and frozen in stock aliquots that were diluted to working concentration (3.4 µmol/L) in medium at the moment of use. Zinc Chloride (ZnCl_2_) was diluted into the cell medium at 100 µM concentration for 16 h. To test proteasome activity, 6 hours before the end of treatment MG132 (Calbiochem, San Diego, CA, USA) was added to the medium at the concentration of 10 µmol/L.

### Cell cultures

Skin Fibroblasts from two non-AD controls (2 females, mean age 63.5±9.2 years) and two AD patients (1 female, 1 male, mean age 66.0±11.3 years) were selected from the cell lines present in our cell repository originally established in 1993 [33]. The diagnosis of probable AD was made according to the criteria developed by National Institute of Neurological and Communicative Disorders and Stroke (NINCDS) and the Alzheimer's Disease and Related Disorders Association (ADRDA). All AD patients presented a 1–4 year history of progressive cognitive impairment predominantly affecting memory. Non-AD patients were without established cognitive disorders. Neither AD nor non-AD patients presented neoplastic diseases at the time of tissue biopsy. All cell lines were frozen at passage 2–4 in a modified growth medium containing 20% foetal bovine serum and 10% dimethylsulfoxide. For the experiments, cell lines were simultaneously thawed and grown up to passages 7–10. Cells were cultured as previously described [21]. Each set of experiments was done using cells at the same passage (ranging from 7 to 10), carefully matching AD and non-AD samples. Culture conditions were kept constant throughout the experiments.

Human embryonic kidney (HEK) 293 cells from European Collection of Cell Cultures (ECACC No. 85120602) were cultured in Eagle's minimum essential medium containing 10% foetal bovine serum, glutamine (2 mM), penicillin/streptomycin (2 mM), at 37°C in 5%CO_2_/95% air [5]. The HEK-293 cells stably transfected with APP751 were obtained as previously described [5] and maintained in G418 at a final concentration of 400 µg/ml.

### Transfection, plasmids and Western immunoblotting

Transient transfection was carried out using the N,N-bis-(2- hydroxyethyl)-2-amino-ethanesulphonic acid-buffered saline (BBS) version of the calcium phosphate procedure and the following plasmids were used: HIPK2-Flag [11] and HIPK2-K1182R (MDM2-resistant) mutant [34]. Total cell extracts were prepared as previously described [20] and immunoblotting was performed with mouse monoclonal anti-Flag (Sigma Chemical Co., St. Louis, MO, USA) and mouse monoclonal anti-tubulin (Immunological Sciences, Rome, Italy). Immunoreactivity was detected by enhanced chemiluminescence kit (Amersham, Little Chalfont, UK).

### p53 conformational immunoprecipitation

p53 conformational state was analyzed by immunoprecipitation as detailed previously [7]. Briefly, cells were lysed in immunoprecipitation buffer (10 mM Tris, pH 7.6; 140 mM NaCl; and 0.5% NP40 including protease inhibitors); 100 µg of total cell extracts were used for immunoprecipitation experiments performed in a volume of 500 µl with 1 µg of the conformation-specific antibodies PAb1620 (wild-type specific) or PAb240 (mutant specific) (Neomarkers, CA, USA). Immunocomplexes were separated by 10% SDS-PAGE and immunoblotting was performed with rabbit anti-p53 antibody (FL393) (Santa Cruz, CA, USA). Immunoreactivity was detected with the ECL-chemiluminescence reaction kit (Amersham, Little Chalfont, UK).

### RNA extraction and reverse transcription-PCR (RT-PCR)

Total RNA was extracted with TRIzol (Invitrogen, Carlsbad, CA, USA) following the manufacturer's instructions. The first strand cDNA was synthesized by reverse-transcribing 5 µg of mRNA with Moloney murine leukaemia virus reverse transcriptase kit and random primers (Applied Biosystems, Foster City, CA, USA). Semiquantitative RT-PCR was carried out by using Hot-Master Taq (Eppendorf, Milan, Italy) with 2 µl cDNA reaction and genes specific oligonucleotides under conditions of linear amplification. DNA products were run on 2% agarose gel and visualized by ethidium bromide staining using UV light. Data presented are representative of at least three independent experiments.

### Chromatin immunoprecipitation (ChIP) analysis

Chromatin immunoprecipitation (ChIP) analysis was carried out essentially as described [13]. Protein complexes were cross-linked to DNA in living cells by adding formaldehyde directly to the cell culture medium at 1% final concentration. Chromatin extracts containing DNA fragments with an average size of 500 bp were incubated overnight at 4°C with milk shaking using polyclonal anti-HIPK2 antibody (Santa Cruz, CA, USA). DNA-protein complexes were recovered with protein G Agarose (Pierce, Rockford, IL, USA). Before use, protein G was blocked with 1 µg/µl sheared herring sperm DNA and 1 µg/µl bovine serum albumin (BSA) overnight at 4°C and then incubated with chromatin and antibodies for 3 hrs at 4°C. PCR was performed using immunoprecipitated DNA and specific primers for human HIF1α and MT2A promoters [15], [17]. Immunoprecipitation with non-specific immunoglobulins (No Ab) was performed as negative controls. PCR products were run on a 2% agarose gel and visualized with ethidium bromide staining using UV light. The amount of precipitated chromatin measured in each PCR was normalized with the amount of chromatin present in the input of each immunoprecipitation.

### Transactivation assay

For transactivation assay HEK-293 and HEK-APP cells were transfected with the p53-target promoter AIP1-luciferase reporter plasmid (kindly provided by H. Arakawa, National Cancer Center, Tokyo, Japan), MT2A-luc (kindly provided by Jean-Mark Vanacker, UMR5242 CNRS/INRA/Université Claude Bernard Lyon/ENS, Lyon, France) or HIF-1α-p800-luc (kindly provided by Carine Michiels, Laboratory of Biochemistry and Cellular Biology, FUNDP-University of Namur, Belgium) reporter plasmids, by using the cationic polymer transfection reagent jetPEI (PolyPlus-transfection, Illkirch, France) according to the manufacturer's instructions. Twenty-four hours later the cells were incubated with 100 µM Zinc Chloride (ZnCl_2_) for 16 h and with 3.4 µM doxorubicin for 24 h before luciferase activity was assayed. Transfection efficiency was normalized with the use of a co-transfected pCMV β-galactosidase plasmid (β-gal). Luciferase activity was assayed on whole cell extract and the luciferase values were normalized to β-gal activity and protein content. At least three independent experiments were performed in duplicate.

### Densitometry and statistics

All the experiments, unless specified, were performed at least three times.

Following acquisition of the Western blot image through an AGFA scanner and analysis by means of the Image 1.47 program (Wayne Rasband, NIH, Research Services Branch, NIMH, Bethesda, MD, USA), the relative densities of the bands were analyzed as described previously [35]. The data were analyzed by analysis of variance (ANOVA) followed when significant by an appropriate post hoc comparison test as indicated in figure legend. The reported data are expressed as means ± SD of at least three independent experiments. A *p* value<0.05 was considered statistically significant.
